# Misinformation and harmful language are interconnected, rather than distinct, challenges

**DOI:** 10.1093/pnasnexus/pgae111

**Published:** 2024-03-12

**Authors:** Mohsen Mosleh, Rocky Cole, David G Rand

**Affiliations:** Department of Management, University of Exeter Business School, Exeter, EX4 4PU, UK; Sloan School of Management, Massachusetts Institute of Technology, Cambridge, MA 02142, USA; Sloan School of Management, Massachusetts Institute of Technology, Cambridge, MA 02142, USA; Sloan School of Management, Massachusetts Institute of Technology, Cambridge, MA 02142, USA; Institute for Data, Systems, and Society, Massachusetts Institute of Technology, Cambridge, MA 02142, USA; Department of Brain and Cognitive Sciences, Massachusetts Institute of Technology, Cambridge, MA 02139, USA

## Abstract

There is considerable concern about users posting misinformation and harmful language on social media. Substantial—yet largely distinct—bodies of research have studied these two kinds of problematic content. Here, we shed light on both research streams by examining the relationship between the sharing of misinformation and the use of harmful language. We do so by creating and analyzing a dataset of 8,687,758 posts from *N* = 6,832 Twitter (now called X) users, and a dataset of *N* = 14,617 true and false headlines from professional fact-checking websites. Our analyses reveal substantial positive associations between misinformation and harmful language. On average, Twitter posts containing links to lower-quality news outlets also contain more harmful language (β = 0.10); and false headlines contain more harmful language than true headlines (β = 0.19). Additionally, Twitter users who share links to lower-quality news sources also use more harmful language—even in non-news posts that are unrelated to (mis)information (β = 0.13). These consistent findings across different datasets and levels of analysis suggest that misinformation and harmful language are related in important ways, rather than being distinct phenomena. At the same, however, the strength of associations is not sufficiently high to make the presence of harmful language a useful diagnostic for information quality: most low-quality information does not contain harmful language, and a considerable fraction of high-quality information does contain harmful language. Overall, our results underscore important opportunities to integrate these largely disconnected strands of research and understand their psychological connections.

## Introduction

In recent years, there has been considerable concern regarding the spread of problematic content on social media. Two types of content that have received particular attention are misinformation (e.g. content that is false, inaccurate, or highly misleading) (1, 2) and harmful language (speech that is insulting, disrespectful, overly negative, or that attacks or diminishes a person or group based on attributes such as race, ethnicity, gender, sexual orientation, nationality, and religion) (3, 4). To date, however, efforts to curb these two kinds of problematic content have proceeded largely in isolation from one another, and the relationship between the two is not well defined or understood.

For the most part, interventions and policies focused on misinformation and harmful language have been developed to narrowly focus on a single type of problematic content. For example, Google's Perspective Application Programming Interface (API) uses machine learning models to identify comments that are “severely toxic,” threatening, insulting, or sexually explicit, among other types of problematic language (5), but notably not misinformation. YouTube has separate hate speech and misinformation policies, suggesting the company considers the two types of problematic content separate phenomena (6, 7). And the “Community Notes” on Twitter (now called X) feature, designed to crowdsource the labeling of online harms, narrowly focuses only on the misleadingness of information and not harmful language; in fact, Twitter deprecated their rating of “harmful” language in 2022 (8).

Here we investigate the extent to which misinformation and harmful language are distinct versus overlapping phenomena. There may be reason to expect misinformation and harmful language to be connected in so much as hateful and toxic posts may use inaccurate or misleading statements about their targets to insult or belittle them; or it could be that posts that seek to mislead others may use harmful language as a persuasive tool (e.g. by making readers more emotional, and thus more susceptible to falsehoods (9)). On the other hand, false or misleading claims need not involve harmful language (e.g. false claims that the 2020 US election was determined by voter fraud); and harmful claims may directly insult and denigrate targets without making (non)factual claims. Beyond the content itself, people who are inclined to spread misinformation may also be inclined to use harmful language: both behaviors are associated with political extremity (e.g. Ref. (2)), and are non-normative (i.e. considered inappropriate by most people).

To evaluate these divergent possibilities, we study 8,687,758 posts from *N* = 6,832 Twitter users. We use classifiers to identify posts involving harmful language, and examine news source links to identify posts with low-quality news. We complement these Twitter data by analyzing harmful language and objective veracity (as judged by professional fact-checkers) in a dataset of *N* = 14,617 true and false headlines.

## Results

**Fig. 1.**
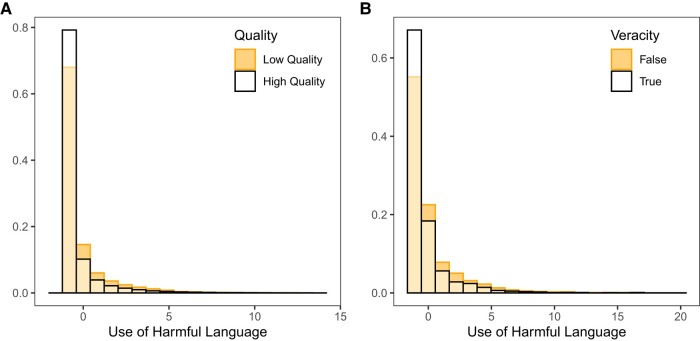
Misinformation is more likely to contain harmful language. a) Distribution of level of harmful language across posts containing links to low-quality websites versus posts containing links to high-quality websites. While our analyses use a continuous news source quality score, for visual clarity in this plot we discretize quality (0–0.5 = low quality, >0.5–1 = high quality). b) Distribution of level of harmful language of headline text across headlines rated as false vs. headlines that rated as true by professional fact-checkers.

All statistics reported in the result section are conducted using linear regression models with standardized coefficients, unless otherwise stated. We begin by comparing the levels of information quality and use of toxic language across posts shared on Twitter (Fig. 1a). Across all posts that contain a link to a news website, linear regression with robust standard errors clustered on user finds a significant negative association between quality of news domain and amount of harmful language (β = −0.102, 95% CI = [−0.122, −0.082], *P* < 0.001; with users characteristic controls: β = −0.095, 95% CI = [−0.115, −0.074], *P* < 0.001). Thus, tweets that contain links to lower-quality news websites also contain more harmful language on average.

**Fig. 2.**
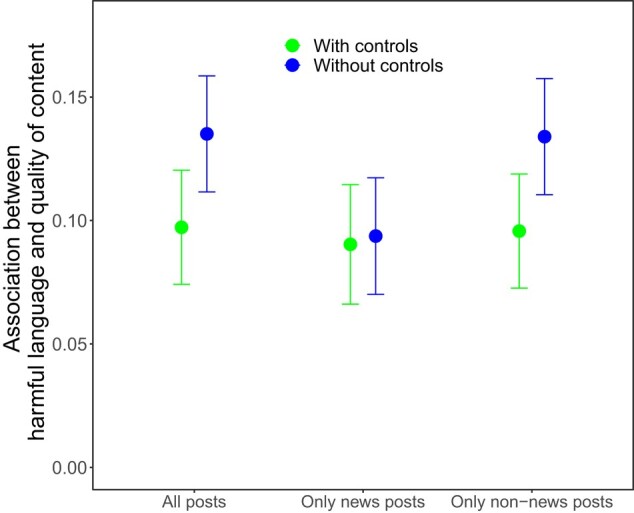
Users who share more-low-quality news domains and use more harmful language. Associations between use of harmful language and quality of content. Shown here are the absolute values of coefficients from linear regression models predicting the use of harmful language using content quality. Error bars indicate 95% CIs.

Next, we do a similar analysis at the user-level, where we predict the average level of use of harmful language across all of the user's posts (whether or not they contain URLs from websites with trustworthiness ratings) using the average quality of news sources the user shared. We find a significant negative association (β = −0.135, 95% CI = [−0.159, −0.112], *P* < 0.001; with users characteristic controls: β = −0.097, 95% CI = [−0.120, −0.074], *P* < 0.001), such that users who use more harmful language on average also share content from less trustworthy websites on average. Importantly, we find similar associations when focusing only on harmful language in the users’ news tweets (β = −0.094, 95% CI = [−0.117, −0.070], *P* < 0.001; with users characteristic controls: β = −0.090, 95% CI = [−0.115, −0.066], *P* < 0.001) and when focusing only on harmful language in the users’ non-news tweets (β = −0.134, 95% CI = [−0.157, −0.110], *P* < 0.001; with users characteristic controls: β = −0.096, 95% CI = [−0.119, −0.073], *P* < 0.001; see Fig. 2). Thus, the user-level association is not simply the result of the tweet-level association documented above, but rather indicates that users who share low-quality news also share (other non-news) content that includes harmful language.

Finally, we complement our analyses of Twitter posts with an analysis of news headlines themselves. To do so, we analyze a set of headlines that were fact-checked by fact-checking website Snopes and Politifact. Linear regression predicting the level of harmful language in the headline text using a dummy variable representing the veracity of the headline provided by the professional fact-checkers finds a significant negative association (β = −0.190, 95% CI = [−0.226, −0.154], *P* < 0.001) which is quite similar to what was observed above in the Twitter data (see Fig. 1b). Thus, false headlines contain more harmful language than true headlines.

## Discussion

Our results demonstrate a link between misinformation and harmful language. Twitter posts containing links to low-quality news outlets are more likely to use harmful language, as are false news headlines. Additionally, users who share more links to low-quality news sources are also more likely to use more harmful language—even in their non-news posts. The similarity in the results we found using Twitter posts and in news headlines is striking and demonstrates the robustness of our findings.

This meaningful association between information quality and harmful language suggests that these two phenomena are significantly related. As a result, there may be important untapped opportunities for synergy between the largely distinct literatures on misinformation and harmful language. Important first steps in such an integration would be further investigation of potential causal mechanisms underlying the association we document. For example, it could be that being hostile and sharing misinformation may have similar psychological roots (e.g. related to status considerations (10)) or it could be that the association is due to a selection effect (similar to toxic people opting into partisan context (11)), where toxic users opt into sharing misinformation. There may also be opportunities for technology companies to develop interventions that simultaneously reduce both misinformation and harmful language, by targeting their intersection.

Importantly, however, although the association between low information quality and harmful language was highly statistically significant, the association was not large enough in magnitude to make harmful language particularly diagnostic for content being misinformation—or to make the content being misinformation particularly diagnostic for it involving harmful language. For example, most low and high-quality posts (and most true and false headlines) used no harmful language. Similarly, many posts and headlines of both kinds did include harmful language. Thus, observing that a post or headline includes harmful language is not sufficient to conclude that it is likely to be misinformation, and observing that a post or headline is inaccurate is not sufficient to conclude that it involves harmful language. This has important implications. It would likely not be productive, for example, to attempt to “inoculate” people against misinformation (12) by describing harmful language as a misinformation technique.

In sum, we present evidence in support of shared psychology between sharing misinformation and the use of harmful language online. Future work should explore the psychological underpinnings of these two related but distinct behaviors.

## Methods

We sampled *N* = 6,832 Twitter users (median 109 followers, 405 following, and 905.5 total posts) randomly selected from those who followed at least 3 major political figures or organizations from Ref. (2). We retrieved all posts from their timelines (up to 3,200 tweets per each user capped by the API rate limit). To measure the quality of content shared by users, we used the reliability of the publisher as a proxy for accuracy of the content via a list of 4,767 news domain trustworthiness ratings (13). To quantify the use of harmful language in tweets, we used ratings from the Google Jigsaw Perspective API (5) and the model from Ref. (14), and then used Principal Component Analysis to reduce the resulting seven ratings to a single harmful language score. For controls, we used the model from Ref. (15) to estimate political affiliation based on political accounts followed by the user, the model from Ref. (16) to estimate the user's probability of being a bot, and the model from Ref. (17) to estimate user gender and age of the user and the probability of the account belonging to an organization; as well as including log number of followers, log number of followings, and log total number of posts (counts are logged due to heavy right-skew). In total, we analyzed 8,687,758 posts of which 198,508 posts contained a link to rated news websites. Our study received a waiver from an ethics review by the MIT Committee on the Use of Humans as Experimental Subjects (COUHES) protocol E-3973.

We also created a dataset of 14,617 headlines scraped from Snopes and PolitiFact, of which 10,672 were false and 3,945 were true; and applied the same approach as above to estimating harmful language use.

See Supplementary material for further methodological details.

## Supplementary Material

pgae111_Supplementary_Data

## Data Availability

Data and code used to generate the results are available at https://osf.io/q5h49/.

## Preprints

This manuscript was posted on a preprint server: https://doi.org/10.31234/osf.io/y5n4u.
