# Supplementary material for: Misinformation and harmful language are interconnected, rather than distinct, challenges
Source: PNAS Nexus. 2024 Mar 12;3(3):pgae111. doi: 10.1093/pnasnexus/pgae111 (PMC10957235; doi:10.1093/pnasnexus/pgae111)
Supplement: pgae111_Supplementary_Data [file pgae111_supplementary_data.pdf]

Supplementary Information

Misinformation and harmful language are interconnected, rather than distinct, challenges

Mohsen Mosleh[1,2]*, Rocky Cole[2], and David G. Rand[2,3,4]

[1]Management Department, University of Exeter Business School, United Kingdom; [2]Sloan School of Management, Massachusetts Institute of Technology, United States; [3]Institute for Data, Systems, and Society, Massachusetts Institute of Technology; [4]Department of Brain and Cognitive Sciences, Massachusetts Institute of Technology, United States.

We collected a sample of Twitter users randomly selected from those who followed at least 3 major political figures or organizations from (2, 18). We retrieved all posts from the timeline of the users (up to 3200 tweets per each user capped by the API rate limit) in December 2022 and extracted domains posted by each user. We also collected all accounts followed by each user.

To measure the quality of content shared by users, we followed a standard practice in the academic literature and used the reliability of the publisher as a proxy for accuracy of content (19-22). Specifically, we used a list of 4,767 news domain trustworthiness ratings, which aggregate across a variety of ratings by professional fact-checkers, journalists, and academics (13). This list contains ratings {0, 0.25, 0.5, 0.75, 1} where lower ratings represent lower quality and misinformation websites.

To quantify the use of harmful language in tweets, we used Google Jigsaw Perspective API (5) and the model from (14). Perspective API provides ratings for "toxicity", "severe toxicity", "identity attack", "insult", "profanity", and "threat" and the model from (14) provides ratings for "hate speech". We winsorized the language features at 99th percentile to reduce the effect of possibly spurious outliers. To capture the most variation across all 7 harmful language measures, we applied Principal Component Analysis (PCA) and used Parallel Analysis to determine the number of retained components. Based on parallel analysis, we used the first Principal Component that represents 59% of variance of all features as a measure for use of harmful language.

Furthermore, we used the statistical model from (15) to estimate political partisanship of the users based on political affiliation of accounts they chose to follow. The idea behind this model is that users on social media platforms are more likely to follow an account that shares a similar political worldview compared to those who do not. Additionally, we used the API service from (16) to calculate the probability of the Twitter account being a bot and used the algorithm from (17) to estimate gender and age of the user and the probability of the account belonging to an organization.

Our sample includes N=6,832 users who shared at least one link from websites for which we have trustworthiness ratings. We analyzed 8,687,758 posts of which 198,508 posts contained a link to rated news websites.

Additionally, we scraped headlines that were fact-checked by two major professional fact-checking websites, namely Snopes and PolitiFact. For headlines we scraped from Snopes, we only included headlines that were rated as “True” or “False”, and for headlines from PolitiFact, we only included headlines that were rated as “True”, “Mostly True”, “Mostly False”, “False”, “Pants on Fire” (we use use binary rating for PolitiFact and classify “True”, “Mostly True” as true; and “Mostly False”, “False”, and “Pants on Fire” as false). This results in a dataset of 14,617 headlines of which 10,672 were false and 3,945 were true (7,016 from Snopes and 7,601 from PolitiFact). Similar to our analyses for the tweets dataset, we applied PCA on ratings for use of harmful language and used the first PC that represents 58% of total variance of the ratings.

All statistics reported in the results are conducted using linear regression models with standardized coefficients, unless otherwise stated. For robustness checks, we include models that have the following control variables: log number of followers, log number of followings, log total number of posts, user estimated political ideology, probability of the account being bot, probability of the account belonging to an organization, user estimated gender, and user estimated age group (a dummy variable for age 18-29, 30-39, 40-above).

Reference list

18. M. Mosleh, D. Rand, Who is on Twitter (“X”)? Identifying demographic of Twitter users. (2024).
19. A. Guess, J. Nagler, J. Tucker, Less than you think: Prevalence and predictors of fake news dissemination on Facebook. *Science advances* **5** , eaau4586 (2019).
20. N. Grinberg, K. Joseph, L. Friedland, B. Swire-Thompson, D. Lazer, Fake news on Twitter during the 2016 US presidential election. *Science* **363** , 374-378 (2019).
21. G. Pennycook *et al.* , Shifting attention to accuracy can reduce misinformation online. *Nature* **592** , 590-595 (2021).
22. M. Mosleh, C. Martel, D. Eckles, D. G. Rand (2021) Perverse Downstream Consequences of Debunking: Being Corrected by Another User for Posting False Political News Increases Subsequent Sharing of Low Quality, Partisan, and Toxic Content in a Twitter Field Experiment. in *proceedings of the 2021 CHI Conference on Human Factors in Computing Systems* , pp 1-13.
